# Cow Farmers’ Homes Host More Diverse Airborne Bacterial Communities Than Pig Farmers’ Homes and Suburban Homes

**DOI:** 10.3389/fmicb.2022.883991

**Published:** 2022-06-17

**Authors:** Hesham Amin, Tina Šantl-Temkiv, Christine Cramer, Ditte V. Vestergaard, Gitte J. Holst, Grethe Elholm, Kai Finster, Randi J. Bertelsen, Vivi Schlünssen, Torben Sigsgaard, Ian P. G. Marshall

**Affiliations:** ^1^Department of Clinical Science, University of Bergen, Bergen, Norway; ^2^Section for Microbiology, Department of Biology, Aarhus University, Aarhus, Denmark; ^3^Department of Public Health, Environment, Work, and Health, Danish Ramazzini Center, Aarhus University, Aarhus, Denmark; ^4^Department of Occupational Medicine, Danish Ramazzini Center, Aarhus University Hospital, Aarhus, Denmark; ^5^The National Research Center for the Working Environment, Copenhagen, Denmark

**Keywords:** cow, pig, dust, airborne bacteria, 16S rRNA gene, indoor environment

## Abstract

Living on a farm has been linked to a lower risk of immunoregulatory disorders, such as asthma, allergy, and inflammatory bowel disease. It is hypothesized that a decrease in the diversity and composition of indoor microbial communities is a sensible explanation for the upsurge in immunoregulatory diseases, with airborne bacteria contributing to this protective effect. However, the composition of this potentially beneficial microbial community in various farm and suburban indoor environments is still to be characterized. We collected settled airborne dust from stables and the associated farmers’ homes and from suburban homes using electrostatic dust collectors (EDCs) over a period of 14 days. Then, quantitative PCR (qPCR) was used to assess bacterial abundance. The V3–V4 region of the bacterial 16S rRNA gene was amplified and sequenced using Ilumina MiSeq in order to assess microbial diversity. The Divisive Amplicon Denoising Algorithm (DADA2) algorithm was used for the inference of amplicon sequence variants from amplicon data. Airborne bacteria were significantly more abundant in farmers’ indoor environments than in suburban homes (*p* < 0.001). Cow farmers’ homes had significantly higher bacterial diversity than pig farmers’ and suburban homes (*p* < 0.001). Bacterial taxa, such as Firmicutes, Prevotellaceae, Lachnospiraceae, and *Lactobacillus* were significantly more abundant in farmers’ homes than suburban homes, and the same was true for beneficial intestinal bacterial species, such as *Lactobacillus amylovorus*, *Eubacterium hallii*, and *Faecalibacterium prausnitzii*. Furthermore, we found a higher similarity between bacterial communities in individual farmers’ homes and their associated cow stables than for pig stables. Our findings contribute with important knowledge on bacterial composition, abundance, and diversity in different environments, which is highly valuable in the discussion on how microbial exposure may contribute to the development of immune-mediated diseases in both children and adults.

## Highlights

- Cow farmers’ homes have higher bacterial diversity than pig farmers’ homes and suburban homes.
- Cow stables have higher bacterial diversity and abundance than pig stables.
- Animal intestinal microbiota appear to contribute to the indoor bacteria in farmers’ homes.
- Putative beneficial bacterial taxa are more abundant indoors in farmers’ homes than in suburban homes.
- Bacterial communities in individual farmers’ homes and cow stables are more similar than pig stables.

## Introduction

Several studies have shown that children growing up on farms have a lower risk of immune-mediated diseases than children growing up in urban areas. These studies link exposure to farm-related microbiota through contact with livestock animals to a lower risk of immunoregulatory disorders, such as allergy, asthma, Irritable Bowel Diseases (IBD), and type 1 diabetes mellitus (Vedanthan et al., 2006; Basinas et al., 2012; Heikkinen et al., 2013; Timm et al., 2014, 2016; Elholm et al., 2016; Stein et al., 2016; Kirjavainen et al., 2019). These observed correlations have contributed to the hygiene hypothesis, which states that low exposure to microorganisms plays a key role in the aetiology of immune-mediated diseases (Okada et al., 2010). Supporting this hypothesis, Ege et al. (2011) found that children who lived on farms were exposed to more diverse environmental microorganisms than the children in the suburban areas.

Microbial dispersal from the farmers working places to the home environment occurs through airflow or direct transport by family members that interact with livestock, soil, water surface, and plants. Thus, the microbial diversity in the home environment may be increased and the composition of the airborne bacterial community in farmers’ homes can be altered compared to suburban homes where humans and, to a lesser extent, pets are the main sources of the indoor air microbiome (Lis et al., 2008; Normand et al., 2011; Hospodsky et al., 2012). Low microbial diversity in urban areas might be the reason for cases of immune dysfunction, poor immune tolerance, and finally may lead to autoimmune disease. However, few studies have characterized the microbial community composition in various farms and farmhouses.

In the current study, we report on results obtained from settled dust from cow stables and cow farmers’ homes collected on an electrostatic dust fall collector (EDC). We compared these results with data obtained using the same approach in pig stables, pig farmers’ homes, and suburban homes (Vestergaard et al., 2018). We focused on the composition, abundance, and diversity of the airborne bacterial communities in cow stables and cow farmers’ homes in comparison to the other indoor environments. The study aimed at (1) comparing type of bacteria present in stables and the farmers’ homes, (2) determining if microbial communities in farmers’ homes differ from suburban homes, and (3) searching for differences in taxonomic groups of putative beneficial bacteria between livestock stables, associated farmers’ homes, and suburban homes.

## Materials and Methods

### Dust Sampling

Electrostatic dust collector was used to collect settled dust from the air with an exposure area of 209 cm^2^ (Juel Holst et al., 2020). Sampling was conducted as part of a previous study in Jutland, Denmark, where settled air dust was collected from the farmers’ homes and associated livestock stables (Vestergaard et al., 2018). Similarly, dust was collected in the suburban homes with the EDCs in the greater Copenhagen area (Juel Holst et al., 2020). During winter (November–April), 25 samples were collected from farmers’ homes and 23 samples from the associated cow stables where the cow farmers were working. During the summer, (May–October), the numbers were 24 and 18, respectively. The EDCs were placed at 1.5 m above the floor, and the sampling period was 14 days. EDCs were kept at −20°C until DNA extraction.

### Dust and DNA Extraction

The EDCs were processed as previously described by Vestergaard et al. (2018). They were carefully placed in a sterile stomacher bag and mixed with 20 ml of extraction buffer, consisting of pyrogen-free water and 0.05% Tween-20. The sample was processed in a stomacher (Star Blender LB 400, Seward, Worthing, United Kingdom) for 10 min at maximum speed. Thereafter, the fluid containing the washed-off dust was transferred to a sterile 50 ml Falcon tube. This procedure was repeated once more, until a total of 40 ml of suspended dust was extracted from the EDC. The dust was collected by centrifugation at 4,700 × *g* for 15 min at 5°C. The supernatant was discarded, and the pellets were resuspended in 1.5 ml of 0.05% Tween-20 extraction buffer. Unexposed EDCs were used for negative control extractions. The dust samples were kept at −20°C until DNA extraction.

The PowerLyzer PowerSoil DNA Isolation kit (MO BIO Laboratories, an Qiagen Company, Germany) was used to extract DNA from the dust pellets following the manufacturer’s instructions with minor modifications including prolonged bead-beating using a TissueLyser bead-beating machine for 2 × 5 min at 50 s^−1^ and prolonged centrifugation steps 13,000 × *g* for 5 min at room temperature following the bead beating step. Negative control extractions were carried out using the same procedures.

### PCR Amplification

Twenty samples from each indoor environment were randomly selected for quantitative PCR (qPCR) to quantify bacterial abundance. Briefly, the qPCR reactions were carried out in a 20 μl reaction volume containing 10 ml SYBR Green 1Master-2x, 2 ml bovine serum albumin (BSA; 10 mg/ml), 1 ml forward primer Bac908F (5′-AAC TCA AAK GAA TTG ACG GG-3′), and 1 ml reverse primer Bac1075R (5′- CAC GAG CTG ACG ACA RCC-3′; 10 pmol/ml; Ohkuma and Kudo, 1998). Controls were obtained by substituting DNA template with ddH2O. Serial dilutions of a plasmid encoding a full-length 16S rRNA gene linked to Sphingomonadales were used to generate standard curves. Thermal cycling and fluorescence measurements were carried out using an MX3005p qPCR machine (Agilent, Santa Clara, CA, United States; RRID:SCR_019526). One cycle of initial denaturation at 95°C for 5 min was followed by 45 cycles of 95°C for 30 s, 56°C for 30 s, 72°C for 20 s, and 80°C for 7 s.

### Illumina MiSeq Sequencing

The 16S rRNA gene was amplified from 114 samples (90 samples, 12 negative control samples, and 12 technical replicates using the same DNA extract). Bac341F (5′-CCT ACG GGN GGC WGC AG-3′) and Bac805R (5′-GAC TAC GGT ATC TAA TCC-3′) bacteria-specific primers were used to amplify V3 and V4 regions (Klindworth et al., 2013). The steps for amplification of the 16S rRNA gene were carried out according to the Illumina protocol (16S Metagenomic Sequencing Library Preparation), with few modifications. The protocol included three PCR steps. In the first PCR, bacteria-specific primers were used to amplify the V3 and V4 regions of the 16S rRNA gene. The PCR mixture containing 2 μl template DNA was used for cow stable samples and 3 μl template DNA was used for farmers’ home samples, 2 × KAPA HiFi Hotstart polymerase (KAPA Biosystems, Wilmington, MA, United States), 0.2 μM forward primer, 0.2 μM reverse primer, and BSA (4 g/L). The variation in the DNA template volume was due to different concentrations of bacteria and PCR inhibitors in the two indoor environments. The thermal cycling was performed in the following steps: an initial denaturation at 95°C for 3 min, 25 cycles with denaturation at 95°C for 30 s, annealing at 55°C for 30 s, elongation at 72°C for 30 s, and a final elongation at 72°C for 5 min. In the second PCR, the Illumina overhang adaptors were added using the same PCR conditions for the first PCR, albeit without added BSA and with only 10 amplification cycles instead of 25. The third PCR Nextera XT Index primers from the Nextera XT Index kit were used. Each reaction contained 2.5 μl Index primer 1 (N7XX) and 2.5 μl Index primer 2 (S5XX), 12.5 μl KAPA HiFi HotStart ReadyMix, and 5 μl dH2O with the same PCR thermal cycling program described above. Following each PCR step, AMPure XP magnetic beads were used for cleaning of the PCR products.

To determine the concentration of the PCR products, the Quant-iTTM dsDNA assay kit and a FLUOstar Omega fluorometric microplate reader (BMG LABTECH, Ortenberg, Germany) were used. Thereafter, the samples were diluted to approximately 3 ng/ml DNA and pooled. The DNA concentrations of pooled samples were measured with a Quant-iTTM dsDNA BR assay kit and on a Qubit fluorometer (Thermo Fisher Scientific, Waltham, MA, United States) before paired end 2 × 300 bp sequenced with a MiSeq sequencer (Illumina, San Diego, CA, United States; RRID:SCR_016379).

### Bioinformatic and Statistical Analysis

The MiSeq-derived sequences from 84 pig farmer homes, 83 pig stables, and 100 suburban homes, along with associated metadata deposited by Vestergaard et al. (2018), were downloaded from the NCBI sequence read archive (SRA) under study number SRP124427. These data were combined with the data obtained in the present study. All sequence data processing, statistical analyses, and visualizations were carried out in RStudio version 1.4.1103 with R version 4.0.4 (R Core Team, 2013).

The sequences were trimmed using the cutadapt package version 1.16 (Martin, 2011) Open-source software package DADA2 version 1.18.0 (Callahan et al., 2016) was used for error correcting and modelling of the sequenced data, mostly by following the tutorial.^1^ One major change was using the shortread package version 1.48.0 (Morgan et al., 2009) to randomly subsample all sequences to 20,000 reads following quality filtering in order to make richness comparisons accurate, as DADA2 tends to inflate richness estimates linearly with an increasing number of reads. Sequences belonging to the forward and reverse read libraries were merged together after primer trimming and quality filtering, and only sequences with a length greater than 430 base pairs were used, which was the expected amplicon length based on the primers. The ASVs were taxonomically classified into species using the DADA2 package’s “assignTaxonomy” and “addSpecies” functions. The reference database used in the current study was the SILVA (RRID:SCR_006423) database version 138 (Quast et al., 2012). To eliminate ASVs contaminating reads in exposed EDC samples, the decontam package version 1.10.0 (Davis et al., 2018) was used. The “prevalence” method was used in the decontam package for contaminant detection. In the prevalence method, the prevalence (presence/absence across samples) of each sequence feature in true exposed EDC sample is compared to the prevalence in negative controls to identify contaminants.

The ampvis2 package version 2.6.8 (Andersen et al., 2018) was used to generate heatmaps and phyloseq version 1.27.6 (McMurdie and Holmes, 2013) and was used to assess the alpha diversity by calculating two diversity measures: observed (the number of individual bacterial taxa) and the Shannon index, which reflects both richness and the relative abundance of each taxon. The Wilcoxon Rank Sum test, implemented in the “wilcox.test” function in R version 4.0.4, was used for the comparison of the alpha diversity indices between different indoor environments as well as to investigate the differences in bacterial abundance measured by qPCR.

Ordination was carried out to compare the microbial communities in different indoor environments, based on the Aitchison dissimilarity matrix calculated using the “dist” function in coda.base package version 0.3.1 (Comas-Cufí, 2020). Principal coordinate analysis (PCoA) was carried out using the ape package version 5.5 (Paradis et al., 2004). Pairwise statistical comparisons were run between different indoor environments using analysis of similarity (ANOSIM) from the vegan package version 2.5-7 (Oksanen et al., 2015) based on the Aitchison dissimilarity matrix.

To identify specific bacterial taxa whose abundances significantly differ between different environmental types, we applied analysis of compositions of microbiomes with bias correction (ANCOM BC) version 1.0.5 (Oksanen et al., 2015). ANCOM BC provides a statistically valid test with a *q* value (adjusted value of *p*) and confidence intervals (log fold change: natural logarithm) for each bacterial taxon. ANCOM BC was performed for bacterial phyla, families, genera, and species levels with a relative abundance equal to or higher than 0.01%. The function “aggregate_taxa” from microbiome package version 1.15.0 (Yang et al., 2022) was used to aggregate taxa to a certain taxonomic level prior to ANCOM BC analysis. For each taxon in the data, ANCOM BC analysis results reported a coefficient value (log fold change) and a *q* value. A negative log fold change indicates that the taxa are less abundant compared to the reference group, and a positive log fold change indicates that the group has a higher abundance compared to the reference group. A *q* value equal to or less than 0.05 indicates a significant difference in abundances of the taxa between the two groups.

The “dist” function in the coda.base package version 0.3.1 was used to construct Aitchison dissimilarity matrix between a farmer’s home and the relevant stable (i.e., the sample pair represents where a farmer lived and worked). The similarity of each pair was ranked among all non-matching home–stable pairs, with the final rank showing how similar associated home–stable pairs were compared to random association between any farmer’s home and any stable. A rank of 1 indicates that there has been substantial bacterial transfer between the farmer’s home and the stable, whereas a random ranking indicates that there has been no link.

### Data Availability

The MiSeq-derived sequences used in this study were deposited in the NCBI under BioProject ID: PRJNA801418.^2^

## Results

### Quality Filtering

Quality filtering and down sampling to 20,000 reads per sample, retain 65 suburban home samples out of 100, 40 cow stable samples out of 41, 38 cow farmers’ homes samples out of 49, 81 pig stable samples out of 83, 82 pig stable samples out of 84, and 43 negative control samples (extraction blank and unexposed EDC samples) out of 52.

### Bacterial Abundance

**Figure 1:**
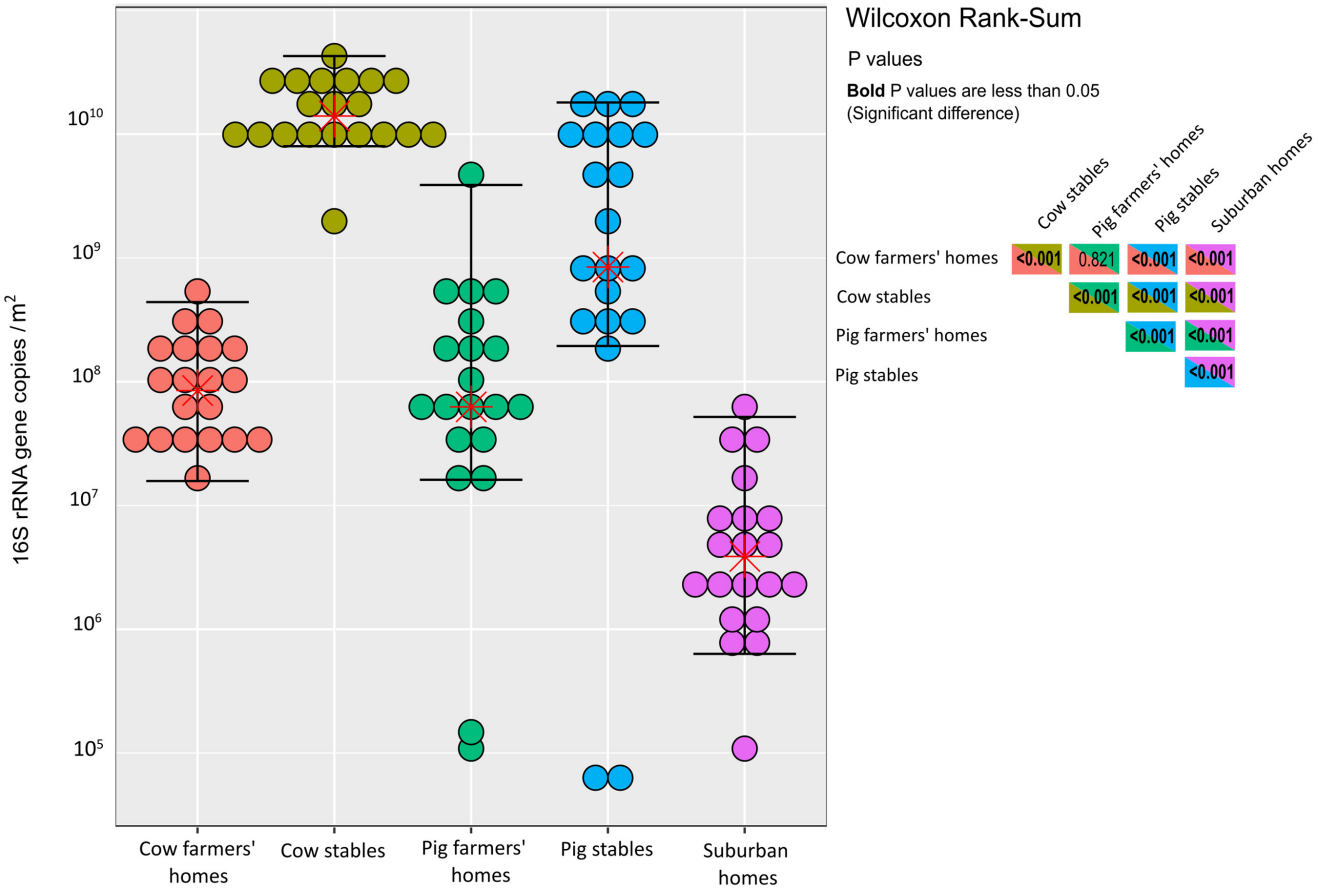
Dot-plot of quantitative PCR measurements of the 16S rRNA genes in each indoor environment. Units are 16S rRNA gene copies per m^2^ of EDC (following 14 days of exposure). The horizontal lines are whiskers of 1.5 IQR (interquartile range) of the upper quartile and lower quartile, while the stars represent the mean. The statistical significance of the differences depicted in this figure is demonstrated in the inset box, which contains Wilcoxon rank sum test results for various comparisons between the indoor environment types. *p* values in bold indicate significance.

We found no significant difference in bacterial abundance between cow and pig farmers’ homes as determined by qPCR (*p* = 0.82; Figure 1). In contrast, there was a significantly higher bacterial abundance in the two types of farmers’ homes compared to the suburban homes (*p* < 0.001), i.e., the total number of airborne bacterial cells was higher in farmers’ homes than in suburban homes, as the observed differences in bacterial abundance were beyond what could be explained solely by copy number variation. Livestock stables showed higher bacterial abundance than all indoor home environments (*p* < 0.001). Cow stables had a higher airborne bacterial load than pig stables (*p* < 0.001) and home environments (*p* < 0.001; Figure 1).

### Alpha Bacterial Diversity

**Figure 2:**
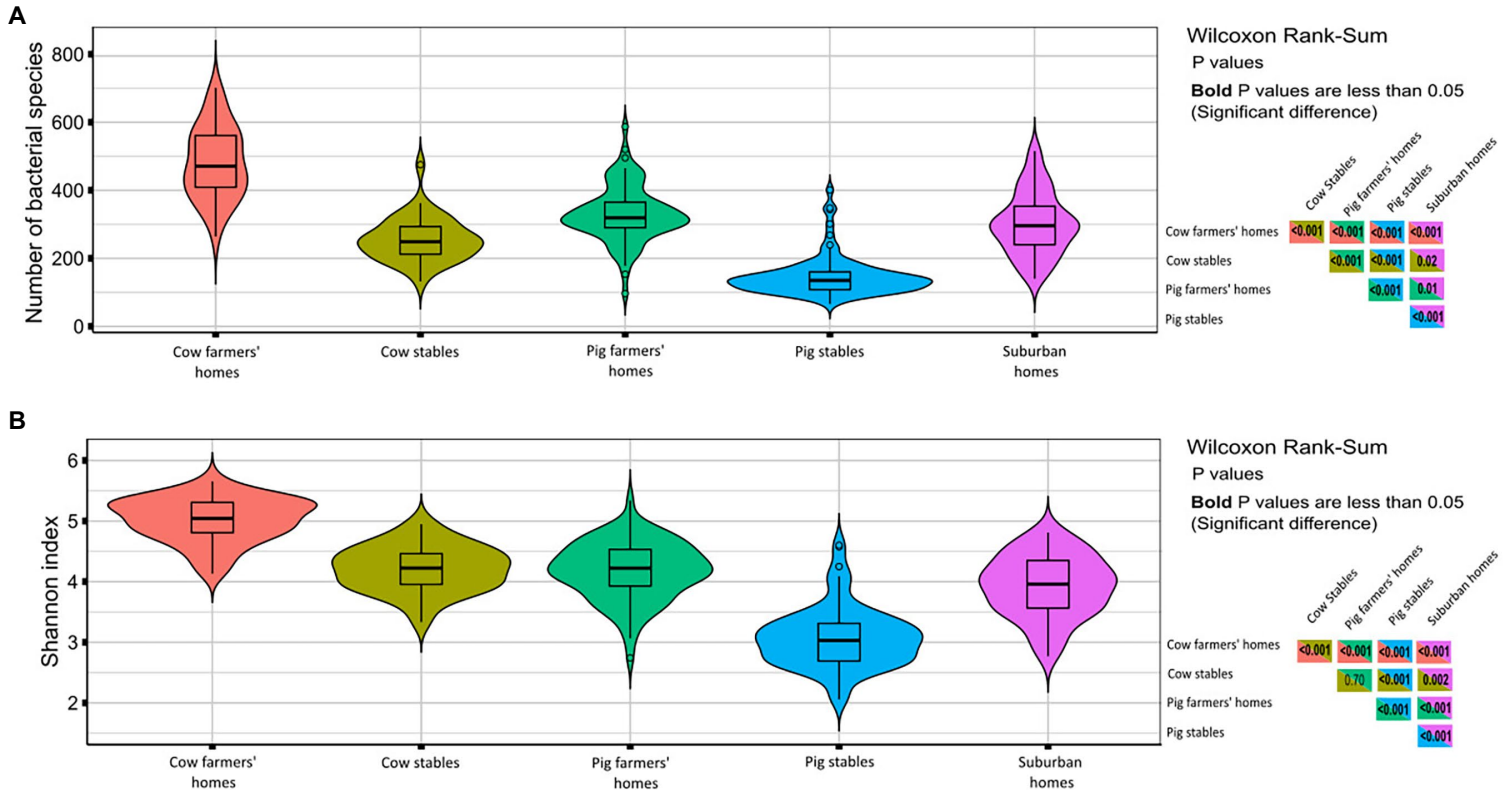
Diversity measures in different indoor environment. The right-hand panels show *p* values from Wilcoxon rank sum test comparing richness/Shannon index in different indoor environments. *p* values in bold are less than 0.05 indicate significant differences. **(A)** Violin plots richness in term of number of bacterial species (OTUs). **(B)** Violin plots of Shannon index considering both the richness and evenness.

In terms of observed richness (numbers of bacterial taxa), the dust from cow farmers’ homes had a significantly higher bacterial richness than pig farmers’ homes and suburban homes (Figure 2A). The livestock stables had lower bacterial richness than farmers’ homes (*p* < 0.001) with the lowest number of bacterial taxa found in dust collected from pig stables (Figure 2A). The Shannon index (a metric for bacterial diversity), which is an estimate of both the richness and uniformity of bacterial communities (Figure 2B), showed the same trend, with cow farmers’ homes having richer and more uniform airborne bacterial community than pig farmers’ and suburban homes (*p* < 0.001) and cow stables harboring a significantly higher bacterial diversity than pig stables (*p* < 0.001).

### Beta Diversity of Indoor Environments

**Figure 3:**
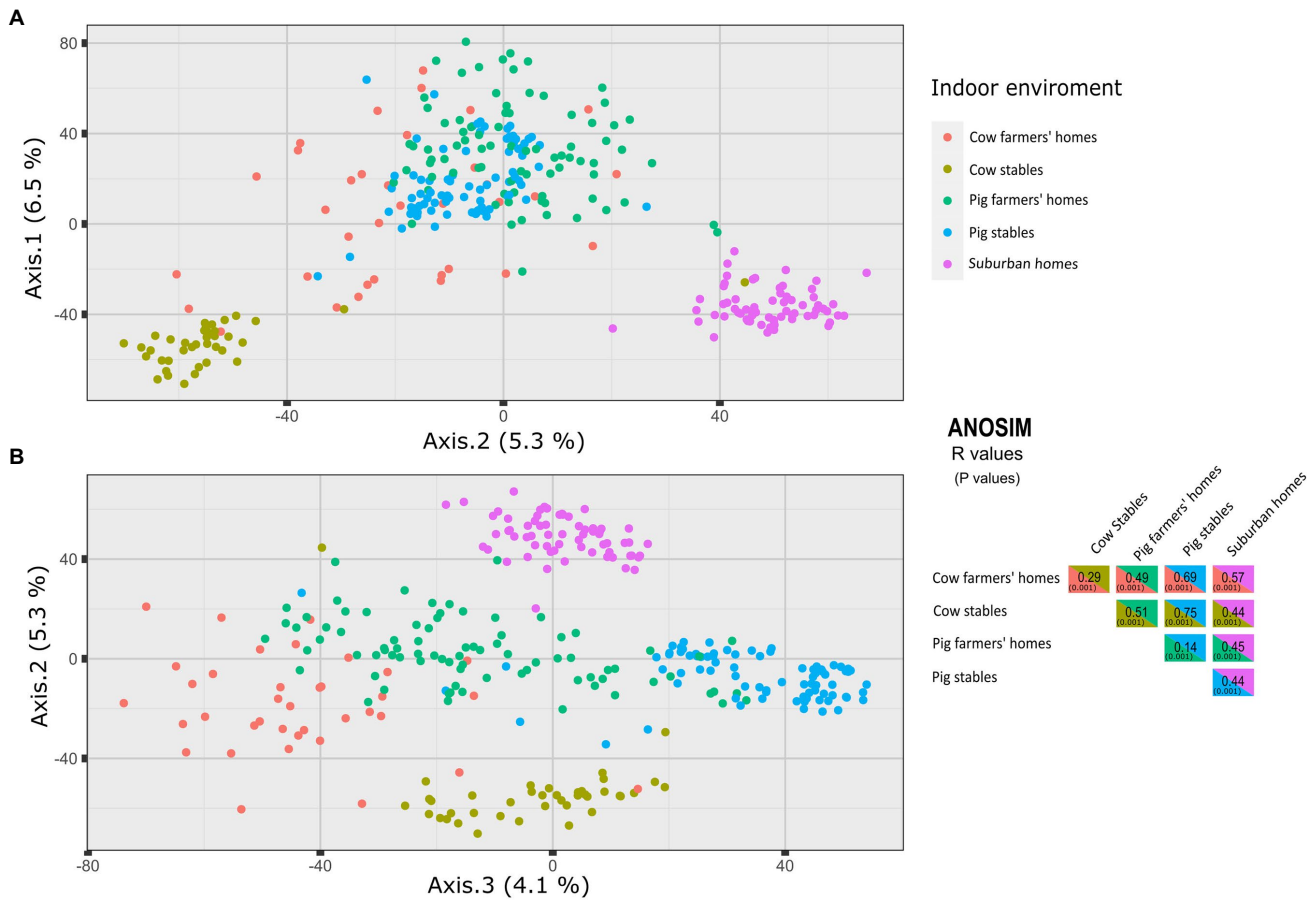
Principal coordinates analysis (PCoA) of axes 1 vs. 2 **(A)**, 2 vs. 3 **(B)** of microbial community structures using Aitchison dissimilarity matrix. The figure shows a significant separation between different indoor environments. On the right side, ANOSIM R metric is used to infer the degree of difference between the environment types, where 1 means very different bacterial communities and 0 means very similar bacterial communities. Values in parentheses are *p* values.

The PCoA using the Aitchison dissimilarity matrix, as well as the ANOSIM test, were used to investigate differences in airborne bacterial composition between different indoor environments. The airborne bacterial communities of farmers’ homes and suburban homes were significantly different based on the ANOSIM test. The PCoA analysis revealed that the microbial community composition of suburban homes clustered separately, while there was a slight overlap between pig and cow farmers’ homes (Figure 3). Despite the overlap between the farmers’ homes, the difference in community composition between cow and pig farmers’ homes was statistically significant (ANOSIM *R* = 0.49, *p* = 0.001). The ANOSIM test also revealed that the difference was greater between cow farmers’ homes and suburban homes (ANOSIM *R* = 0.57, *p* = 0.001) compared to pig farmers’ homes and suburban homes (ANOSIM *R* = 0.45, *p* = 0.001). The microbial community composition was more similar between pig stables and pig farmers’ homes (ANOSIM *R* = 0.14, *p* = 0.001) than between cow stables and cow farmers’ homes (ANOSIM *R* = 0.29, *p* = 0.001). The largest pairwise difference across all indoor environments was observed for pig and cow stables (ANOSIM *R* = 0.75, *p* = 0.001). All distinctions between the different indoor environments are visible in the spatial organization of samples plotted using PCoA (Figure 3).

### Seasonal Effects on Bacterial Community Composition and Abundance

The effect of season on the indoor airborne bacterial community in the cow farmers’ home and cow stables was limited. Season had no significant effect on bacterial load (Supplementary Figure 1) or bacterial community composition (Supplementary Figure 2). Bacterial diversity and richness of cow stables were not affected by the season, whereas bacterial richness (number of bacterial taxa) was significantly higher in cow farmers’ homes in the summer compared to the winter (*p* = 0.03; Supplementary Figure 3).

### Bacterial Community Composition

**Figure 4:**
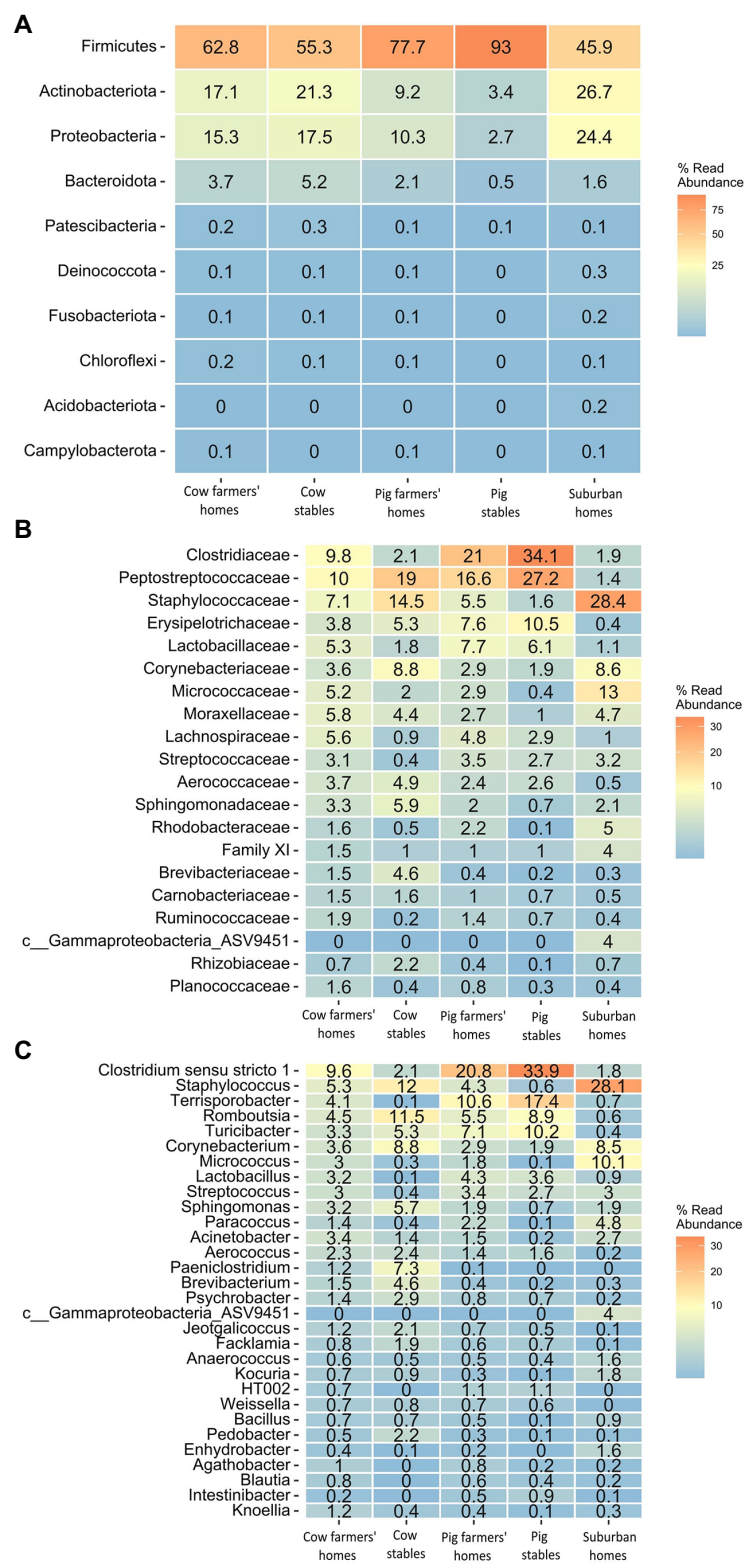
Heatmap indicating community-level composition, number indicating percentage (mean value of relative abundance) of bacterial taxa in different indoor environment. **(A)** Top 10 bacterial phyla. **(B)** Top 20 bacterial families. **(C)** Top 30 bacterial genera.

Almost all samples were dominated by four bacterial phyla independent of the sampling location: Firmicutes, Proteobacteria, Actinobacteria, and Bacteroidetes (Figure 4A). Firmicutes were more abundant in pig farmers’ homes than in any other indoor home environment. Proteobacteria and Actinobacteria were the most prevalent in suburban homes, with 24.4 and 26.7%, respectively. Bacteroidetes were found in greater abundance in cow farmers’ homes than in pig farmers’ or suburban homes. These tendencies in phylum abundance were found to be significant by ANCOM BC analysis, except in the case of Bacteroidetes abundance (Supplementary Tables 1–3). Members of the Firmicutes families, Lachnospiraceae, Lactobacillaceae, Ruminococcaceae, and Peptostreptococcaceae were significantly more abundant in the farmer’s homes than in the suburban homes. Apart from Peptostreptococcaceae, the other three families were relatively more abundant in farmers’ homes than in livestock stables (Figure 4B). However, not all of them were significant between farmers’ homes and stables (Supplementary Tables 4, 5). Rikenellaceae and Prevotellaceae families that belong to the Bacteroidetes phylum were found to be more abundant in farmers’ homes than in suburban homes. Rikenellaceae, Prevotellaceae, Peptostreptococcaceae, and Lachnospiraceae were significantly more abundant in pig farmers’ homes than in cow farmers’ homes. However, Ruminococcaceae and Lactobacillaceae did not show a significant difference in abundance between the two types of farmers’ homes (Supplementary Tables 4, 5). The Gram-positive bacterial families, such as Staphylococcaceae, Corynebacteriaceae, Micrococcaceae, and Streptococcaceae dominated the airborne microbial communities in suburban homes (Figure 4B; Supplementary Table 6).

On the genus level, bacterial genera associated with the animal gut, including *Lactobacillus*, *Turicbacter*, *Intestinibacter*, *Terrisporobacter*, *Lachnospiraceae* UCG-007, and *Romboustia* based on AMDB: database of animal gut microbial communities (Yang et al., 2022), were found to be significantly more abundant in pig stables, followed by the pig farmers’ home, than in any other indoor environment, including cow stables (Figure 4C). Some of these bacterial genera, such as *Lactobacillus and Turicbacter*, did not show a significant difference between the cow farmers’ homes and suburban homes (Supplementary Tables 7–9). Species level identification also showed several bacterial species of intestinal origin (Yang et al., 2022), such as *Eubacterium hallii*, *Faecalibacterium prausnitzii*, *Lactobacillus amylovorus*, and *Clostridium butyricum* to be significantly more abundant in farmers’ homes than in suburban homes. Apart from *Clostridium butyricum*, all the above-mentioned bacterial species were found to be significantly more abundant in farmers’ homes than in livestock stables (Supplementary Tables 10–12). Bacterial species that were associated with animal and livestock environments, such as *Saccharopolyspora rectivirgula*, *Staphylococcus sciuri*, and *Streptococcus suis* were found in significantly greater abundance in associated farmers’ homes than in suburban homes (Supplementary Tables 10–12).

### Similarity of Bacterial Community Between the Farmer’s Home and the Associated Stable

We performed a pairwise analysis of similarity between bacterial communities in livestock stables and associated farmer’s homes (i.e., the pair of locations where the farmer worked and lived) to investigate whether the two associated indoor environments were more similar to each other than was the general similarity between unassociated stables and farmers’ homes. We found that nine out of 29 (31%) associated cow stable–cow farmer’s home pairs were more similar than non-associated stable–home pairs (Supplementary Figure 4). While we found only 14 out of 77 (18%) associated pig stable–pig farmers’ home pairs had substantial bacterial transfer (Supplementary Figure 5). The quantitative pairwise distance values between the home and stables pairs are shown in Supplementary Tables 13–16. These values show that the number of shared bacteria between farmers’ homes and cow stables is higher than between farmers’ homes and pig stables. However, in general, the indoor air bacterial community in a specific farmer’s home was more likely to be similar to the indoor air bacterial community in another farmer’s stable than to the indoor air bacterial community in his or her own stable.

## Discussion

In this study, we investigated the airborne bacterial communities of five indoor environments: pig and cow farmers’ homes, suburban homes, and pig and cow stables. The results demonstrate that the abundance, alpha and beta diversity, and community composition of airborne bacteria differ significantly between farmers’ and suburban homes. We also showed that the gut microbiome of the farm animals contributed to the indoor airborne bacterial communities in farmers’ homes, especially in the case of pig farmers’ homes.

### Higher Indoor Airborne Bacterial Abundance in Farmers’ Home Than Suburban Homes

We found significantly higher bacterial abundance in cow and pig farmers’ homes than in suburban homes (Figure 1), as previously reported by Pakarinen et al. (2008). In rural areas, a greater variety of outdoor microbial sources such as plants, soil, and livestock animals might explain the higher prevalence of microbes in farmers’ homes compared to suburban homes. Bacterial abundance was 10–100 times higher in livestock stables than in the home environments (Figure 1). Increased bacterial abundance in livestock stables is consistent with prior research that found higher bacterial abundance in livestock-stable air compared to other indoor environments (Dungan et al., 2011; Hong et al., 2012). Aerosolization of dust particles and bacteria associated with animal skin and faces might explain the increase in bacterial abundance in livestock stables compared to home environments. According to Wei et al. attachment of airborne bacteria to the dust particles increases their viability, abundance, and metabolic capability thus alter the fate of bacterial cells in the air due to protection by the dust particles from harsh environmental conditions such as stables (Hu et al., 2020).

Cow stables exhibited a larger bacterial abundance in the air than pig stables. We cannot exclude the possibility that the difference in bacterial load could be due to differences in the number of animals as well as the design of the two types of livestock stables. Kembel et al. (2012) showed that natural ventilation significantly increased bacterial abundance compared to mechanically ventilated indoor environments. In the present study, the natural ventilation in the cow stable compared to mechanical ventilation in the pig stable may also have contributed to the difference in bacterial abundance.

### Cow Farmers’ Homes Have a High Level of Alpha Bacterial Diversity

The difference in bacterial richness and bacterial diversity (Shannon index) between the three home environments was significant. We demonstrate for the first time that cow farmers’ homes have the highest bacterial diversity compared to the pig farmers’ homes and suburban homes (Figure 2). The difference in airborne bacterial diversity between the two types of farmers’ homes and suburban homes could be attributed to bacterial exposure from their livestock stables and bacterial dispersal from the outdoors to the indoor environment through ventilation. Lis et al. (2008) found that airborne microorganisms in farmers’ homes consisted of a mixture of microorganisms from farm buildings, including livestock stables. They suggested that bacteria and fungi are transported from farm buildings to homes *via* workers’ clothes and bodies. Pasanen et al. (1989) also concluded that airborne microorganisms may be indirectly transmitted from cow stables to farmhouses *via* workers’ clothing. We speculate that the higher bacterial diversity and richness in cow farmers’ homes compared to pig farmers’ homes might be due to the microbes from pig and humans being more similar than those of cows and humans due to a more similar diet for pigs and humans compared to cows and humans. Another explanation could be because Danish regulations require farmers’ working in pig stables to wash their hands and change clothes to prevent the spread of zoonotic pathogens from pigs are more strict compared to farmers working in open-air and less precautioned cow stables, so cow farmers would transmit more bacteria to their homes than pig farmers (Denver et al., 2016).

Seasonal differences in airborne bacterial richness but not bacterial diversity in cow farmers’ homes were significant (Supplementary Figure 3). We found higher bacterial richness in the summer compared to the winter. This could be due to the fact that Denmark, where samples were collected, is located in Northern Europe, and experiences a temperate climate. Because air-conditioning systems are not common, people normally ventilate their homes by opening windows, as they would do more in summer when central heating is not running. Thus, it would bring in more bacteria taxa from the surrounding environment, which would lead to a rise in bacterial richness. In addition, the diversity of outdoor bacterial communities in Scandinavia was found to be higher in the summer compared to winter (Karlsson et al., 2020).

Increased bacterial diversity in the indoor environment has been linked to a lower prevalence of immunoregulatory disorders, including IBD, atopy, asthma, and type 1 diabetes mellitus. Timm et al. (2014) discovered that being born and living on a livestock farm for the first 5 years of life was associated with a lower risk of IBD when compared to being born and living in the city. It was hypothesized by the authors that the association could be due to decreased microbial diversity (Timm et al., 2014). Exposure to a variety of microorganisms has been inversely associated with the risk of asthma and atopy (Ege et al., 2011). Similarly, Valkonen et al. (2015) found that bacterial diversity was inversely related to atopy but not asthma. Others have suggested that exposure to a broad variety of non-pathogenic environmental microorganisms during childhood might have a protective effect against type 1 diabetes mellitus (Heikkinen et al., 2013). Our finding supports the different putative health outcomes between different indoor environments based on different levels of bacterial diversity.

Our results show that the bacterial diversity is higher in cow stables as compared to pig stables. The natural ventilation in cow stables, as opposed to the mechanical ventilation and highly controlled, closed nature of the pig stables, might explain the increased indoor airborne bacterial diversity in the cow stables compared to pig stables, where the bacteria in the air will mainly come from a limited source, the pigs, and their feed. Other factors that could explain higher bacterial diversity in cow stables compared to pig stables include reduced use of antibiotics in cow farming compared to pig farming. Illi et al. (2012) reported that cow exposure is the farm exposure that protects against asthma and atopy. In the same study, the exposure to pigs did not show a protective effect against asthma and atopy (Illi et al., 2012).

### Beta Diversity of the Indoor Airborne Bacterial Communities

According to the PCoA analysis, suburban homes had a distinct bacterial community, while the pig and cow farmers’ homes showed a minor overlap (Figure 3). Different indoor bacterial community composition between suburban and farmers’ homes is in line with previous studies, which showed a difference in microbiota between farm and non-farm homes (Kirjavainen et al., 2019; Fu et al., 2021). Even though there is an overlap in the community composition of cow and pig farmers’ homes, there was a significant difference in the community composition between cow and pig farmers’ homes. As a result, it is feasible that the putative protective effects of airborne microbiomes in the cow and pig farmers’ homes might be different. The microbial communities were more similar between pig stables and pig farmers’ homes than between cow stables and cow farmers’ homes (Figure 3). As Stein et al. (2016) showed, distance to home and farmers’ work might be an important factor explaining this similarity, but this information was not available in our study.

The PCoA analysis and ANOSIM showed that pig and cow stables had different indoor bacterial community compositions. This indicates that farmers working in these environments (stables) have different microbial exposure and, therefore, may experience different health consequences. Several studies report an inverse relationship between animal contact and the prevalence of atopy and respiratory allergy in childhood (Naleway, 2004; Vedanthan et al., 2006). In Denmark, Elholm et al. (2013) showed that exposure to farm animals protects against the development of atopy not only in childhood but also in young adulthood. They found that being exposed to cows, pigs, or combinations of these animals was associated with a decreased risk of new-onset sensitization when compared to participants without livestock exposure. The exposure to endotoxin has been associated with a reduced prevalence of sensitization to common allergens in a highly exposed adult farming (Portengen et al., 2005). In Alpine farm environments, the GABRIEL Study found that children who were exposed to cows, but not pigs, were protected against asthma, atopic sensitization, and hay fever (Illi et al., 2012). The lower number of pigs per farm in the alpine region compared to Jutland, Denmark, might explain why the association differs between the two studies with regard to exposure to pigs.

### Indoor Airborne Bacterial Composition Between Rural and Suburban Areas

All samples, regardless of the environmental type, were dominated by four bacterial phyla: Firmicutes, Proteobacteria, Actinobacteria, and Bacteroidetes. This is largely consistent with prior studies that showed the predominance of the four bacterial phyla in cow and pig stables and indoor home environments (Hong et al., 2012; Boissy et al., 2014; Zhang et al., 2019). The higher abundance of Firmicutes and lower abundance of Proteobacteria in farmers’ homes relative to suburban homes is basically in line with prior epidemiological findings suggesting that Firmicutes decrease the risk of atopic sensitization (Lee et al., 2021). In contrast, Proteobacteria have been associated with allergy and found to be more common in the airways of neutrophilic asthma patients (Yang et al., 2018). Bacterial families that might have protective effects against allergy, IBD, and asthma were found to be significantly more abundant in farmers’ homes than in suburban homes. These include members of the Firmicutes families, Lachnospiraceae, Lactobacillaceae, Ruminococcaceae (Lynch et al., 2014; Huang and Boushey, 2015), and Peptostreptococcaceae (Pekkanen et al., 2018). With the exception of Peptostreptococcaceae, the other three families that were suggested to have a protective effect against autoimmune disease were relatively more abundant in farmers’ homes than in livestock stables, implying that the surrounding outdoor environment in rural areas could be the source of these bacterial families. This is consistent with the suggestion by Dimich-Ward et al. that some aspects of the protective effect of the farm environment are not attributable to contact with livestock (Dimich-Ward et al., 2006).

Rikenellaceae and Prevotellaceae families that belong to the Bacteroidetes phylum were found to be more abundant in farmers’ homes, especially cow farmers’ homes, than in suburban homes. Members of these two families are frequently found in cattle’s gastrointestinal microbiota (Mao et al., 2015). Rikenellaceae and Prevotellaceae have been associated with protection against allergic asthma and allergy, with a possible explanation that the inhaled or ingested bacteria serve as a kind of an anti-allergy adjuvant for the allergens inhaled or ingested, a concept supported by recent research showing commensal bacteria protect against food allergen sensitization (Huang and Boushey, 2015).

We found beneficial taxa of gut microbiome to be more abundant in farmers’ homes, especially pig farmers’ homes, than in suburban homes. Recent animal and epidemiological studies have found that certain bacterial taxa have protective effects against inflammation, IBD, insulin resistance, and atopy (Radman et al., 2015; Udayappan et al., 2016; He et al., 2021). Oral treatment of diabetic mice with *Eubacterium hallii* leads to an improvement in insulin sensitivity (Udayappan et al., 2016). *Faecalibacterium prausnitzii* was discovered to boost the secretion of IL-10 thereby inhibit the creation of proinflammatory cytokines, such as TNF-α, IL-6, and IL-12 (He et al., 2021). Another bacterial species that showed higher relative abundance in farmers’ homes than in suburban homes was *Lactobacillus amylovorus*. Using intestinal human and intestinal pig cells as substrate, *L. amylovorus* was able to be inhibit the TLR4 (Toll-like receptors) inflammatory signaling *via* modulation of TLR2 and cytokine regulation (Finamore et al., 2014).

The majority of the bacterial species mentioned above were significantly more abundant in farmers’ homes than in livestock stables. This could indicate that the environment surrounding farmers’ homes might be the source of these bacterial taxa. The animal manure used in fields as fertilizer where farmers’ homes are located might be a source for the presence of animal gut microbiota in the indoor air of the farmers’ homes.

### Bacterial Transfer From Livestock Stables to the Farmers’ Homes

Overall, the microbial communities were more similar between pig stables and pig farmers’ homes (ANOSIM *R* = 0.14) than between cow stables and cow farmers’ homes (ANOSIM *R* = 0.29; Figure 3). However, we found a higher similarity in bacterial communities established in individual farmers’ homes and their associated cow stables than pig stables, indicating more bacterial transfer from the cow stable than pig stables to the associated farmers’ homes. Lower rate of bacterial transfer rate between pig stables and their associated farmer homes were previously reported and discussed by Vestergaard et al. (2018). It seems at first paradoxical that the airborne bacterial communities in pig farmers’ homes are generally more similar to pig stables, while individual cow farmers’ homes are more similar to their corresponding stables, but if we separate the concepts of similarity and transfer between stable and home then it makes more sense. It means that there is some general property of pig stables or pig farmers’ homes (perhaps the porcine microbiota is more similar to the human than the bovine?) that makes them more similar but given microbial community data from a specific cow stable one would be more likely to accurately pair it to a specific cow farmer’s home than for pigs, which is a separate concept.

Higher similarity in bacterial communities established in individual farmers’ homes and their associated cow stables than for homes and pig stables. This could be a result of the strict Danish regulations that require employees present in pig stables to disinfect their hands, change clothes, and disinfect equipment to prevent the transmission of zoonotic diseases from pigs compared to less pre-cautioned and open-air cow stables (Denver et al., 2016).

In most cases, the indoor airborne bacteria in the farmers’ homes did not originate from the cow or pig stables where they were working. This might imply that putatively beneficial bacteria in the farmers’ homes air are transported from outdoor sources in the environment surrounding the farmers’ homes rather than from the farmers’ own pig or cow stables. This is consistent with the findings of Dimich-Ward et al., who suggested that some aspects of the farm environment, other than contact with livestock, were protective of respiratory and allergic conditions (Dimich-Ward et al., 2006). Outdoor environmental sources are responsible for increasing bacterial diversity in farmers’ homes compared to suburban homes. Among these potential sources are plants, soil, water, and pig manure. In Denmark, pig manure is commonly used as a low-cost natural fertilizer for agricultural soil to increase crop yield and maintain soil fertility (Sommer and Knudsen, 2021). We found several bacteria taxa related to gut microbiota to be significantly more abundant in farmers’ homes than in suburban homes (Figure 4C). This suggests an indirect transfer of microbes from the gut of the pigs to the indoor air of the farm home, which might explain the putatively beneficial bacteria common in the air of both farmers’ homes and pig stables.

## Conclusion

The settled airborne dust in farmers’ homes, especially cow farmers’ homes, was characterized by high bacterial diversity compared to suburban homes that were dominated by bacteria from human sources and had low bacterial diversity. Furthermore, intestinal animal microbiota from manure appears to contribute to the indoor airborne bacterial community in farmers’ homes. All the observed differences in bacterial community composition, diversity, and abundance of specific types of bacteria found in this study support the concept that the bacterial composition in farmers’ homes, and to a lesser extent, livestock stables, further contribute to a verification of the microbial diversity hypothesis. Further studies, including experimental animal models and immunological studies, are needed to demonstrate the possible beneficial effects of specific bacterial taxa, which are abundant in rural environments.

## Data Availability Statement

The datasets presented in this study can be found in online repositories. The names of the repository/repositories and accession number(s) can be found in the article/Supplementary Material.

## Author Contributions

GE, VS, and TS designed and carried out sample collection from cow stables and farmers’ homes under the SUS12 study. DV, GH, KF, TS, TŠ-T, and IM planned qPCR and 16S rRNA gene sequencing. DV and GH extracted cells from EDC filters. DV extracted DNA from cells and carried out PCR and sequencing. HA and IM analyzed the data from sequencing and qPCR. HA, RB, IM, and TS wrote the manuscript. All authors contributed to the article and approved the submitted version.

## Funding

This work was funded by Realdania and by the Danish National Research Foundation (grant no. DNRF104). Additionally, this project has received funding from the European Research Council (ERC) under the European Union’s Horizon 2020 research and innovation program (grant agreement no. 804199).

## Conflict of Interest

The authors declare that the research was conducted in the absence of any commercial or financial relationships that could be construed as a potential conflict of interest.

## Publisher’s Note

All claims expressed in this article are solely those of the authors and do not necessarily represent those of their affiliated organizations, or those of the publisher, the editors and the reviewers. Any product that may be evaluated in this article, or claim that may be made by its manufacturer, is not guaranteed or endorsed by the publisher.

## Footnotes

^1^ benjjneb.github.io/dada2/tutorial_1_8

^2^ https://www.ncbi.nlm.nih.gov/sra/PRJNA801418

## Supplementary Material

Supplementary Figure 1 Dot-plot of quantitative PCR measurements of cow farmers’ homes and cow stables based on season. The 10 dots in the dot-plot represents the results of 10 measurements. The horizontal lines are whiskers of 1.5 IQR of the upper quartile and lower quartile The significance of the differences depicted in this figure is demonstrated in the inset box, which contains Wilcoxon rank sum test results. P values less than 0.05 is significant.

Supplementary Figure 2 Principal coordinates analysis (PCoA) of microbial community structures of cow farmers’ homes and cow stables based on season using Aitchison dissimilarity matrix. On the right side ANOSIM R metric is used to infer the degree which the environment, where 1 means very different communities and zero means very similar communities. A significant difference (P < 0.05).

Supplementary Figure 3 Diversity measures of cow farmers’ homes and cow stables based on season. P values from Wilcoxon rank sum test comparing richness/Shannon index in different indoor environments. P values in bold are less than 0.05 indicate significant differences. **(A)** Violin plots richness in term of number of bacterial species (OTUs). **(B)** Violin plots of Shannon index considering both the richness and evenness.

Supplementary Figure 4 Plots showing the similarity rank of associated cow stable — cow farmers’ home pairs in a ranked list of all possible cow stable-home pairs. A similarity rank of 1 (Green bar) indicates that the airborne bacterial community in a given cow farmer's home is more similar to the cow stables where that farmer works than any other cow stable. Summer samples pairs showed 3 out of 12, while winter showed 6 out of 17 pairs were more similar than non-associated stable–home pairs.

Supplementary Figure 5 Plots showing the similarity rank of associated pig stable — pig farmers’ home pairs in a ranked list of all possible pig stable-home pairs. A similarity rank of 1 (Green bar) indicates that the airborne bacterial community in a given pig farmer's home is more similar to the pig stables where that farmer works than any other pig stable. Summer samples pairs showed 10 out of 40, while winter showed 4 out of 37 pairs were more similar than non-associated stable–home pairs.

## Abbreviations

ASVs: Amplicon sequence variants
DADA: Divisive Amplicon Denoising Algorithm
EDC: Electrostatic dust collector
IBD: Irritable Bowel Diseases
qPCR: Quantitative PCR
