# Supplementary figures and images for: Cow Farmers’ Homes Host More Diverse Airborne Bacterial Communities Than Pig Farmers’ Homes and Suburban Homes
Source: Front Microbiol. 2022 Jun 17;13:883991. doi: 10.3389/fmicb.2022.883991 (PMC9278274; doi:10.3389/fmicb.2022.883991)

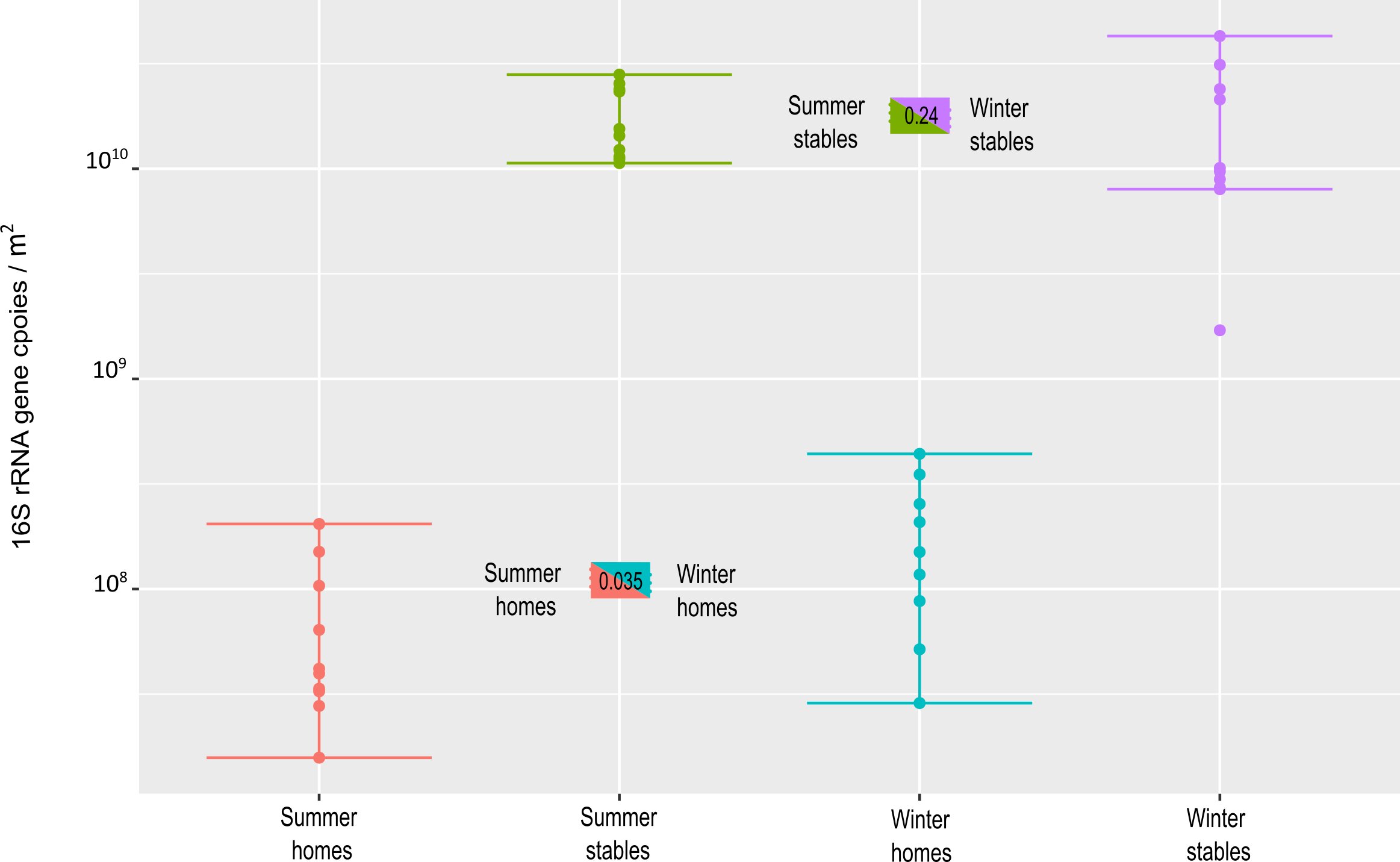

Supplement: Supplementary Figure 1 — Dot-plot of quantitative PCR measurements of cow farmers’ homes and cow stables based on season. The 10 dots in the dot-plot represents the results of 10 measurements. The horizontal lines are whiskers of 1.5 IQR of the upper quartile and lower quartile The significance of the differences depicted in this figure is demonstrated in the inset box, which contains Wilcoxon rank sum test results. P values less than 0.05 is significant. [file Image_1.jpg]

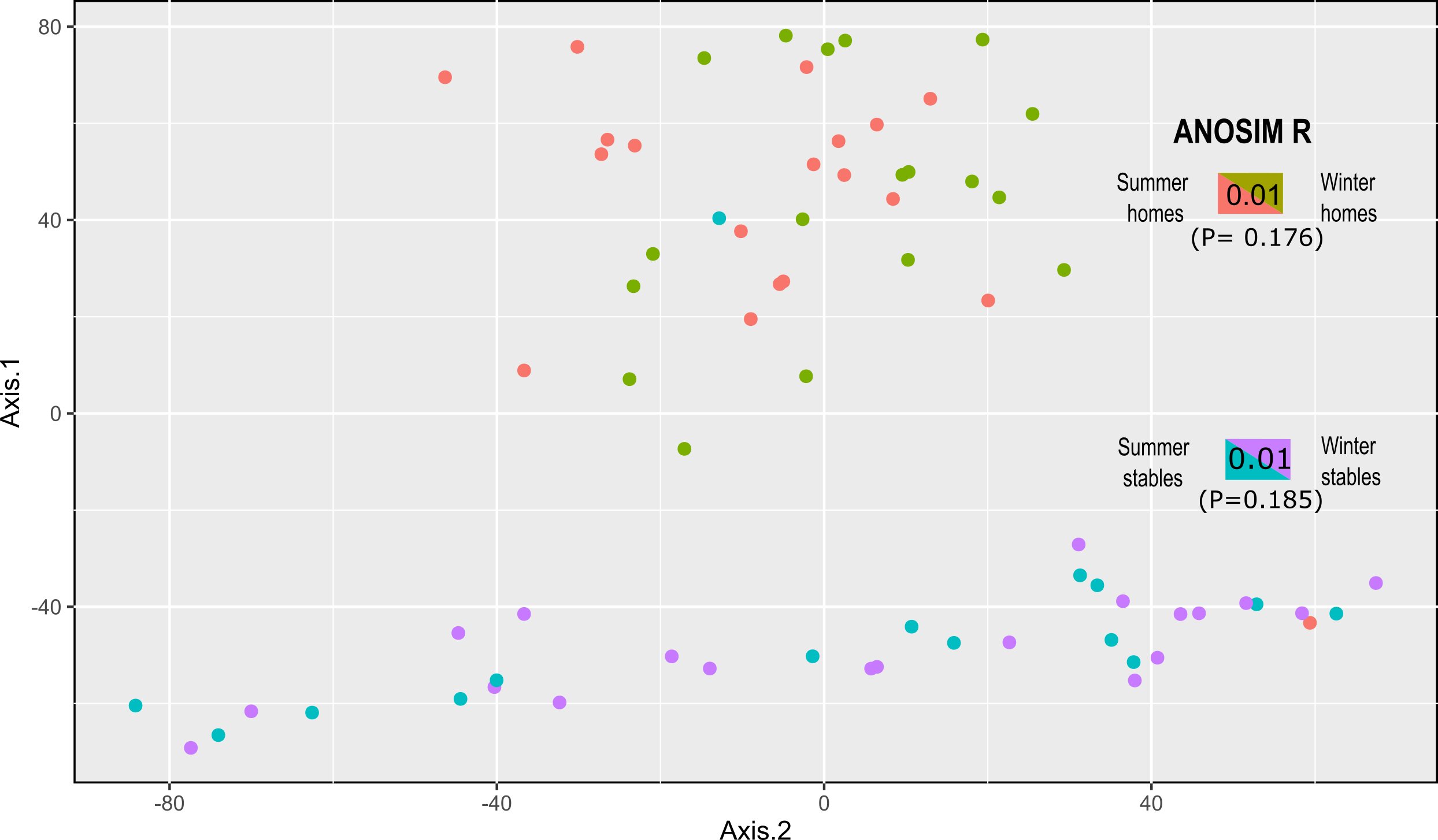

Supplement: Supplementary Figure 2 — Principal coordinates analysis (PCoA) of microbial community structures of cow farmers’ homes and cow stables based on season using Aitchison dissimilarity matrix. On the right side ANOSIM R metric is used to infer the degree which the environment, where 1 means very different communities and zero means very similar communities. A significant difference (P < 0.05). [file Image_2.jpg]

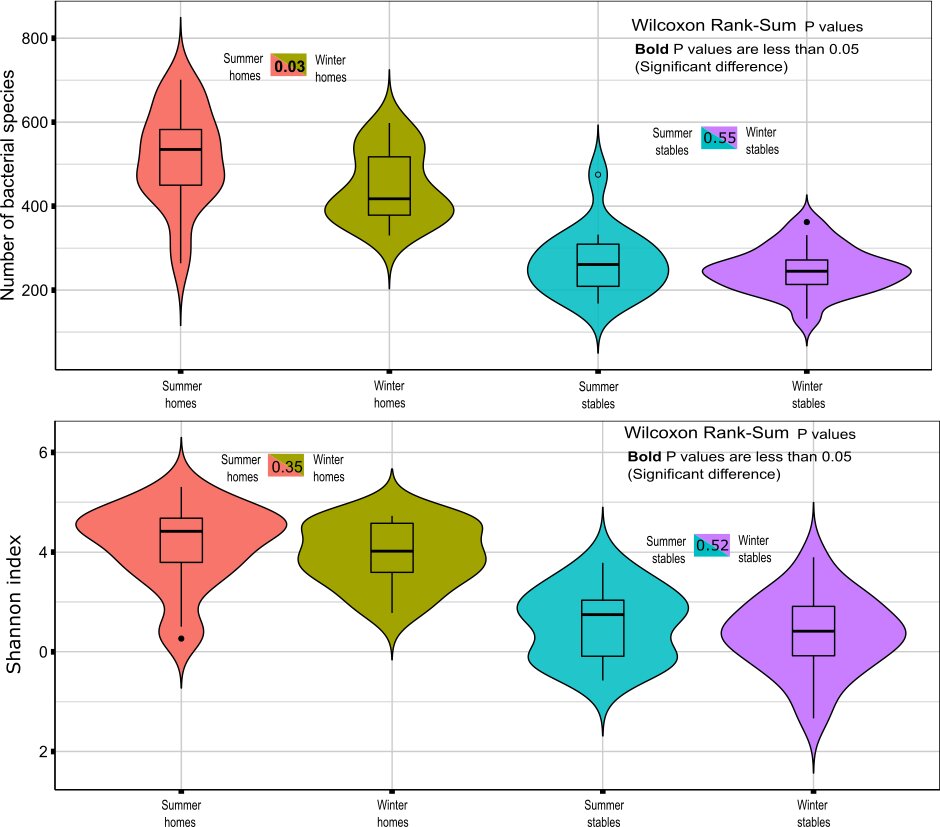

Supplement: Supplementary Figure 3 — Diversity measures of cow farmers’ homes and cow stables based on season. P values from Wilcoxon rank sum test comparing richness/Shannon index in different indoor environments. P values in bold are less than 0.05 indicate significant differences. (A) Violin plots richness in term of number of bacterial species (OTUs). (B) Violin plots of Shannon index considering both the richness and evenness. [file Image_3.jpg]

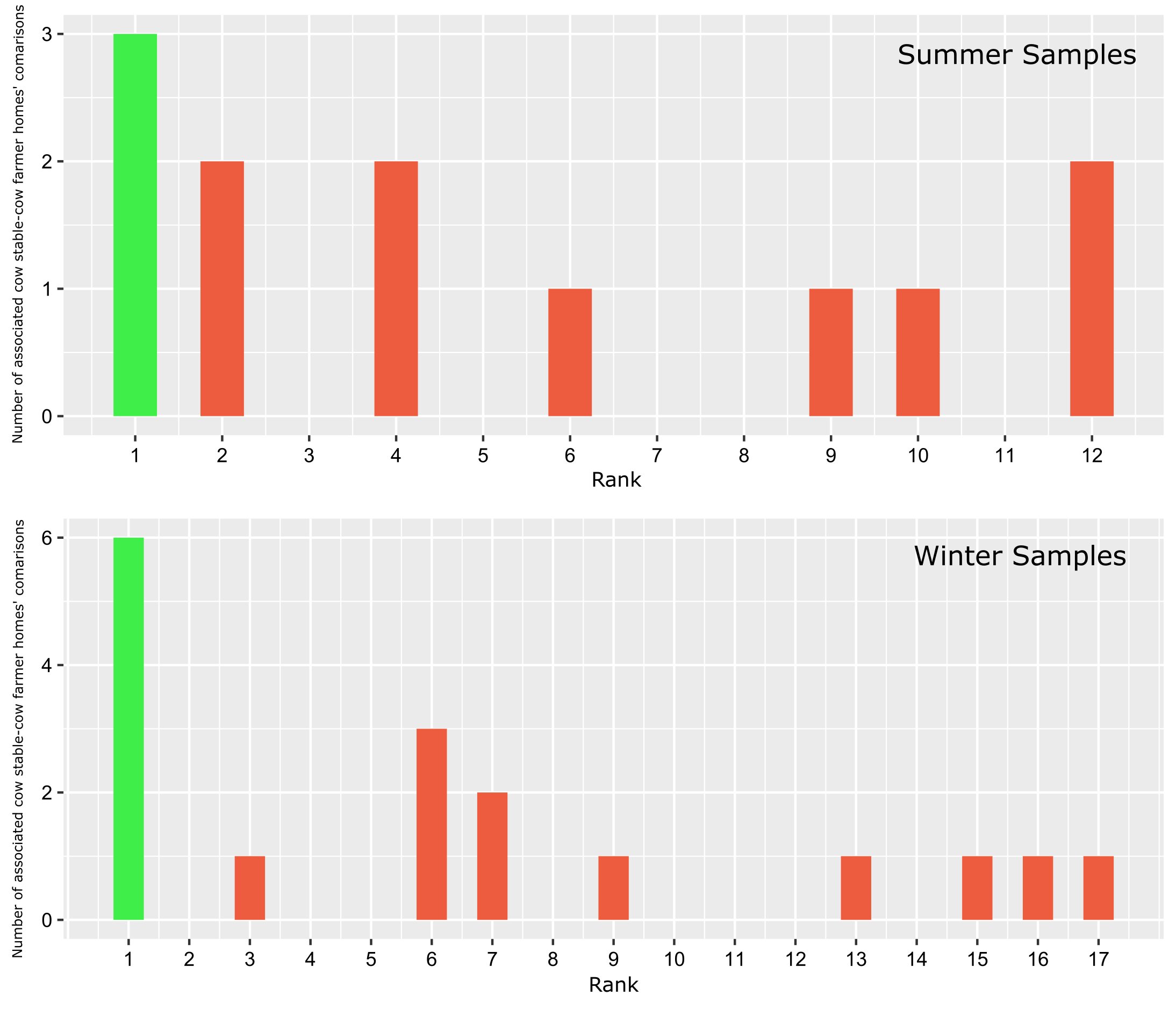

Supplement: Supplementary Figure 4 — Plots showing the similarity rank of associated cow stable — cow farmers’ home pairs in a ranked list of all possible cow stable-home pairs. A similarity rank of 1 (Green bar) indicates that the airborne bacterial community in a given cow farmer's home is more similar to the cow stables where that farmer works than any other cow stable. Summer samples pairs showed 3 out of 12, while winter showed 6 out of 17 pairs were more similar than non-associated stable–home pairs. [file Image_4.jpg]

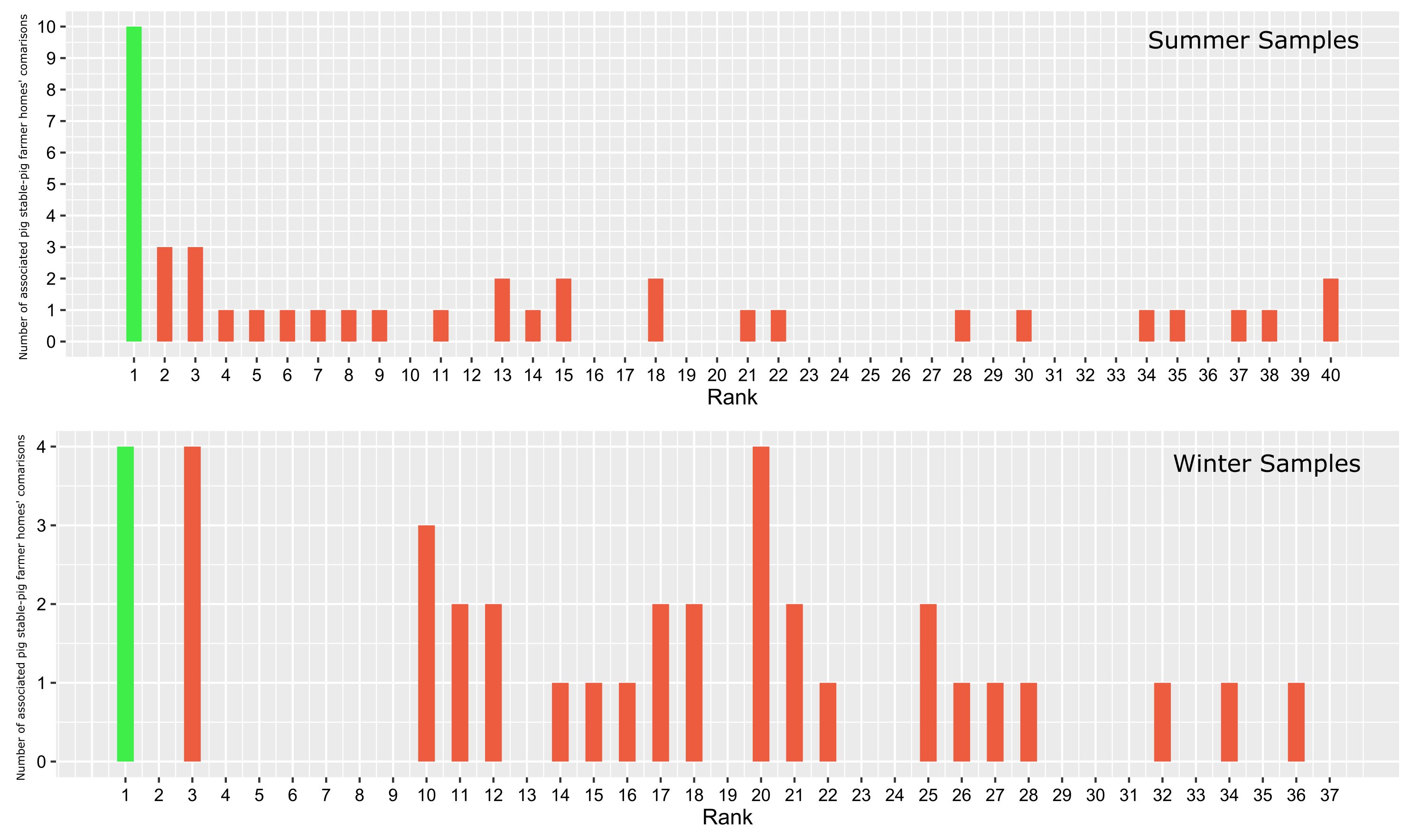

Supplement: Supplementary Figure 5 — Plots showing the similarity rank of associated pig stable — pig farmers’ home pairs in a ranked list of all possible pig stable-home pairs. A similarity rank of 1 (Green bar) indicates that the airborne bacterial community in a given pig farmer's home is more similar to the pig stables where that farmer works than any other pig stable. Summer samples pairs showed 10 out of 40, while winter showed 4 out of 37 pairs were more similar than non-associated stable–home pairs. [file Image_5.jpg]
